# Supplementary material for: Mesenchymal stem cells exert renoprotection via extracellular vesicle-mediated modulation of M2 macrophages and spleen-kidney network
Source: Commun Biol. 2022 Jul 28;5:753. doi: 10.1038/s42003-022-03712-2 (PMC9334610; doi:10.1038/s42003-022-03712-2)
Supplement: Supplementary file 2 — Supplementary Information [file 42003_2022_3712_MOESM2_ESM.pdf]

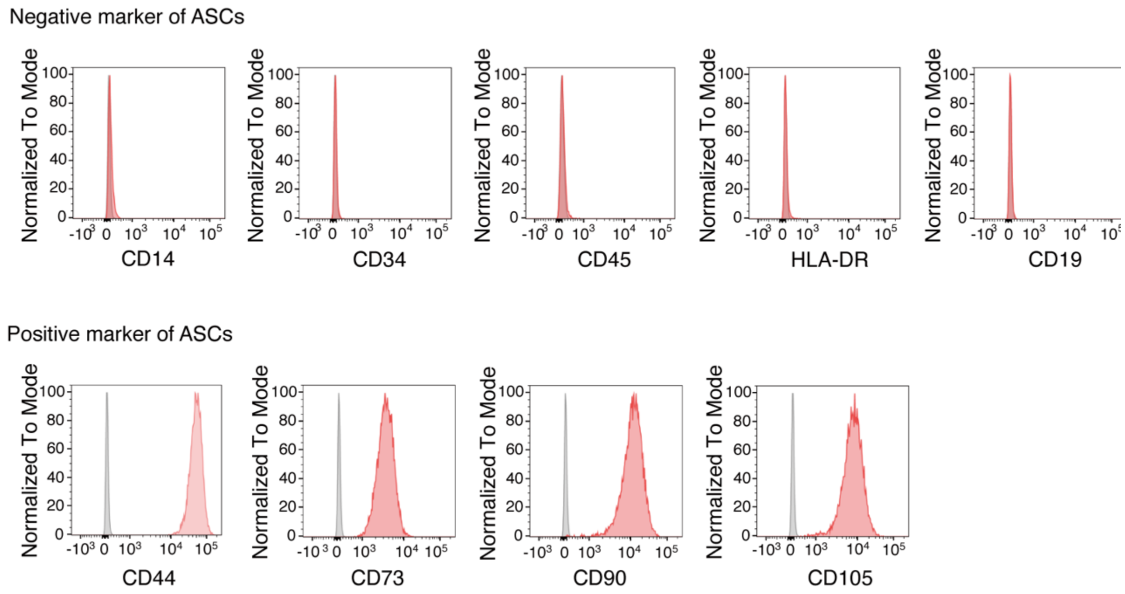

Supplementary Figure 1. Expression analysis of typical cell surface markers on ASCs.

ASCs were negative for the antigens CD14, CD34, CD45, HLA-DR, and CD19 but positive for the antigens CD44, CD73, CD90, and CD105.

Supplementary Figure 2

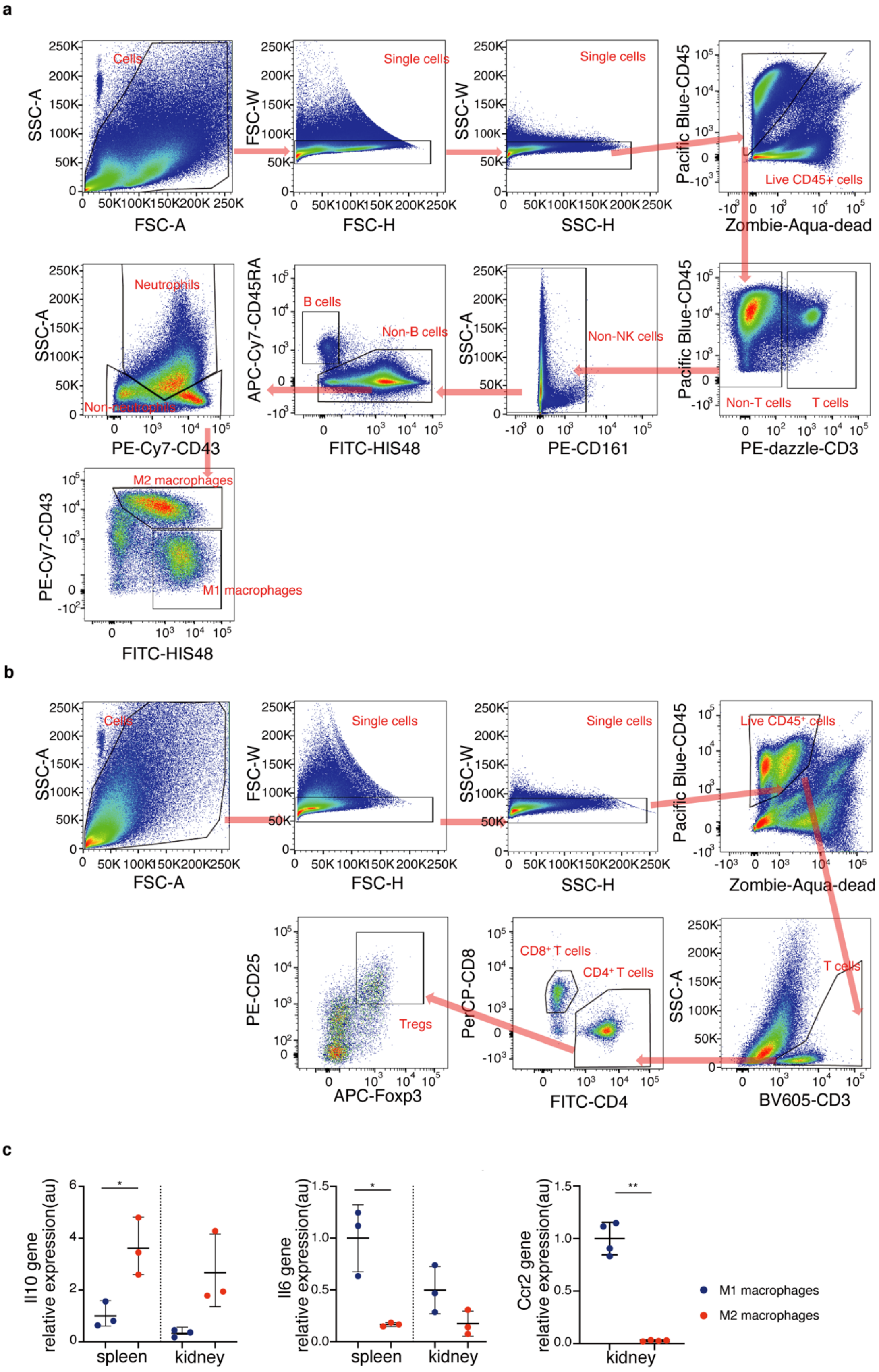

Supplementary Figure 2. Flow cytometric panels for leukocyte subsets.

(a) Gating strategy used for flow cytometric analysis of neutrophils, M1 macrophages, M2 macrophages, and B cells. (b) Gating strategy used for flow cytometric analysis of CD4+ T cells, CD8+ T cells, and Tregs. (c) mRNA expression levels of IL-10 and IL-6 in M1 and M2 macrophages in the spleen and kidney (n=3 per group), and mRNA expression of CCR2 in M1 and M2 macrophages in the kidney (n=4 per group). All data are shown as mean ± SD. *$p \leq 0.05$, **$p \leq 0.01$ as determined by Welch's t-test.

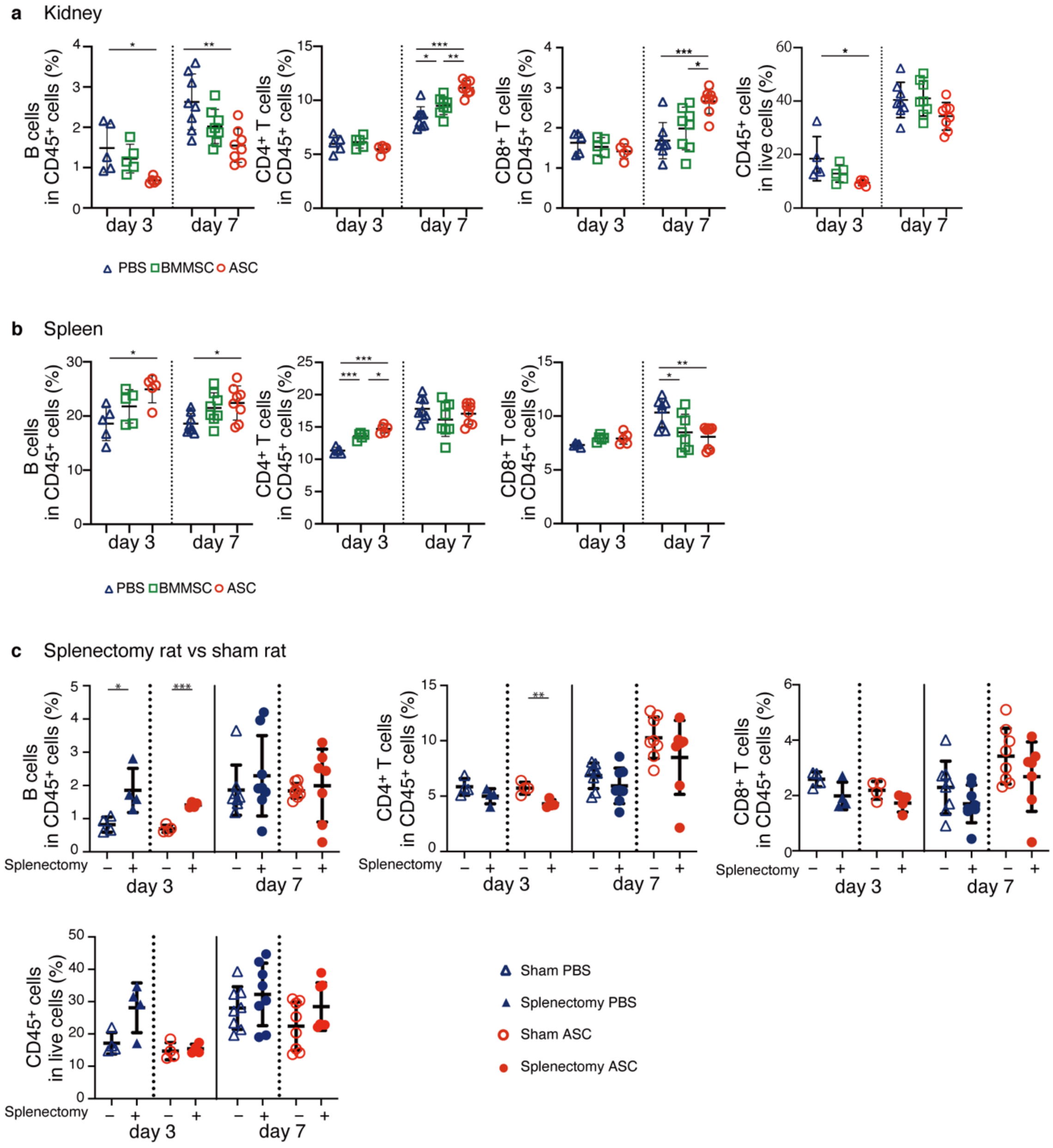

Supplementary Figure 3. Flow cytometric analysis of leukocyte subsets.

(a) Frequency of B cells, CD4+ T cells, CD8+ T cells in CD45+ cells, and CD45+ cells in live cells in the kidney 3 and 7 days after nephritis induction (day 3, n=5 per group; day 7, n=8 per group). *$p \leq 0.05$, **$p \leq 0.01$, ***$p \leq 0.001$ as determined by ANOVA. (b) Frequency of B cells, CD4+ T cells, and CD8+ T cells in CD45+ cells in the spleen 3 and 7 days after nephritis induction (day 3, n=5 per group; day 7, n=8 per group). *$p \leq 0.05$, **$p \leq 0.01$, ***$p \leq 0.001$ as determined by ANOVA. (c) Alterations in the percentage of B cells, CD4+ T cells, CD8+ T cells in CD45+ cells, and CD45+ cells in live cells induced by splenectomy with and without ASC treatment for nephritis (day 3, n=4 per group; day 7, n=7-8 per group). All data are shown as mean ± SD. *$p \leq 0.05$, **$p \leq 0.01$, ***$p \leq 0.001$ as determined by Welch's t-test.

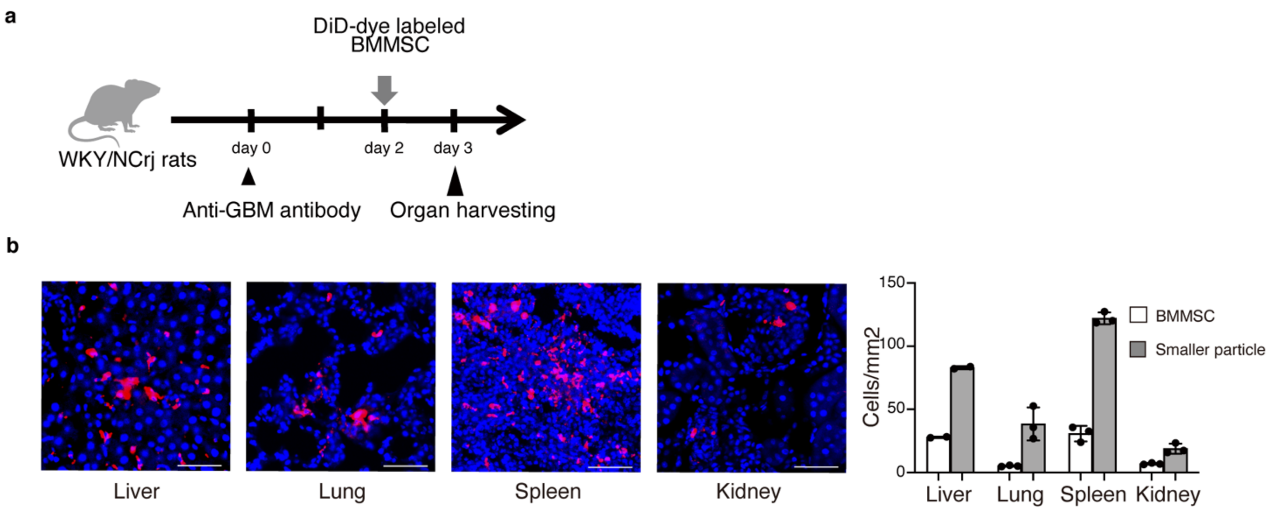

Supplementary Figure 4. DiD-positive cells were abundant in the lungs and spleen after DiD-labeled BMMSC administration.

(a) Experimental scheme for analyzing BMMSC distribution. DiD dye-labeled BMMSCs were administrated to rats with nephritis on day 2. Tissues were harvested on day 3. (b) Number of DiD-positive cells with large DiD dye particles and small particles in the liver, lung, spleen, and kidney were counted. (Liver: n=2; lung, spleen, and kidney: n=3). All data are shown as mean ± SD. Scale bars, 100 μm.

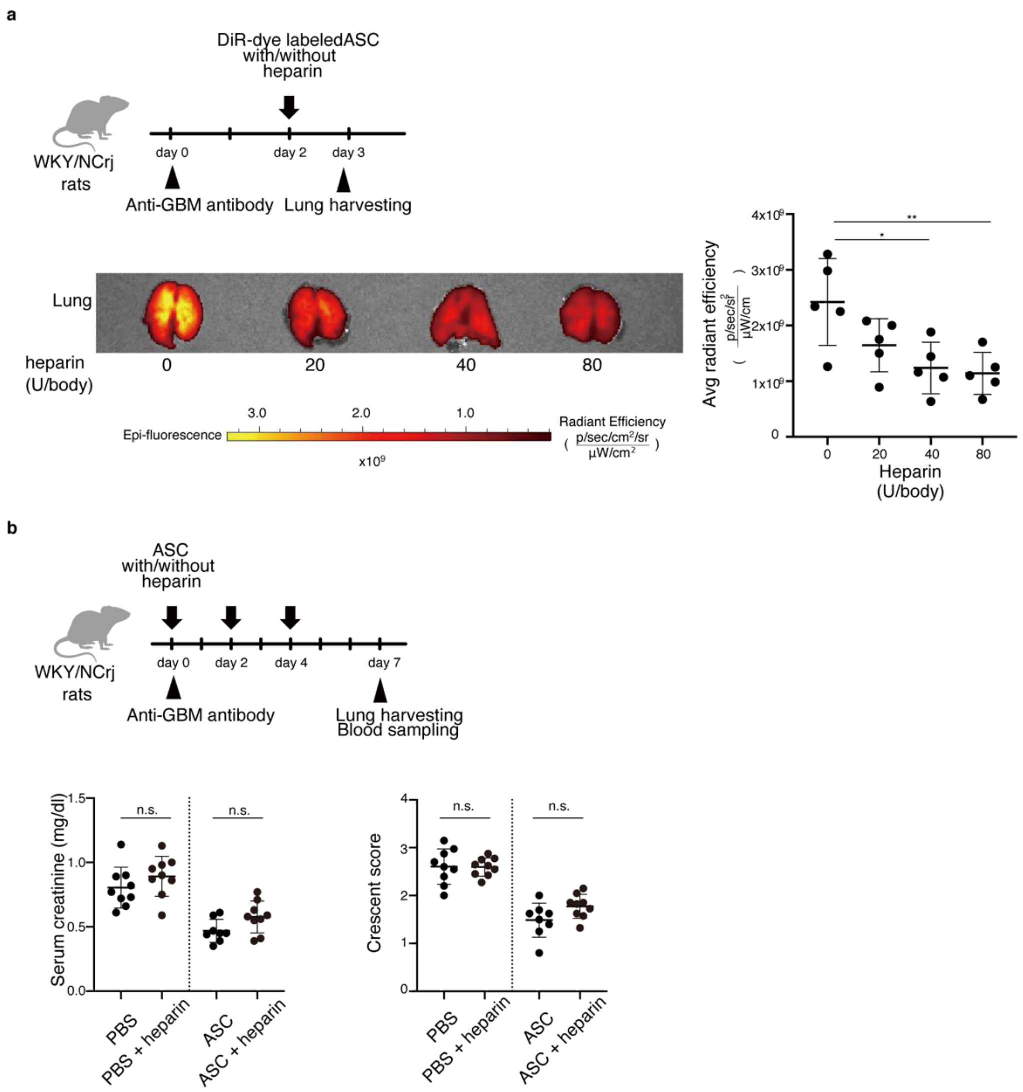

Supplementary Figure 5. Impact of the reduction of ASC accumulation in the lung on treatment efficacy.

(a) Experimental scheme for IVIS imaging analysis. DiR dye-labeled human ASCs were administered to nephritis-induced WKY/NCrj rats on day 2 with and without heparin. IVIS observation was performed on day 3. The accumulation of ASCs in the lungs was decreased by using heparin in a dose-dependent manner (n=5 per group). (b) Experimental scheme for ASC treatment with or without heparin. The therapeutic potential of ASCs was not canceled by decreasing the accumulation of ASCs in the lungs (n=8–9 per group). All data are shown as mean ± SD. *$p \leq 0.05$, **$p \leq 0.01$, as determined by ANOVA.

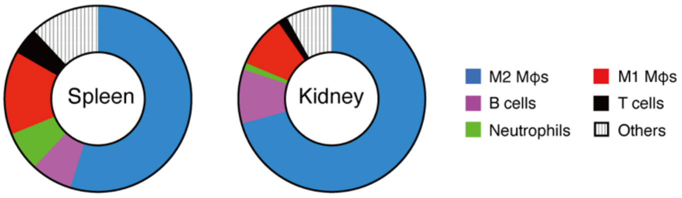

Supplementary Figure 6. The proportion of each subset in DiD$^+$ leucocytes.

Pie charts show the proportion of each subset in DiD$^+$ leukocytes in the spleen and the kidney 4 hours after the venous injection of DiD-labeled ASCs (n=6 per group).

Supplementary Table 1. Summary of MSigDB gene set enrichment analyis

		DEG_group_1			DEG_group_2			DEG_group_3			DEG_group_4			DEG_group_5			DEG_group_6			DEG_group_7		
Term	K	k	p_value	FDR_q_value	k	p_value	FDR_q_value	k	p_value	FDR_q_value	k	p_value	FDR_q_value	k	p_value	FDR_q_value	k	p_value	FDR_q_value	k	p_value	FDR_q_value
HALLMARK_GLYCOLYSIS	200	4	0.00431	0.0239	8	6.69E-07	1.11E-05	NA	NA	NA	NA	NA	NA	3	0.00759	0.0237	4	0.00279	0.00734	NA	NA	NA
HALLMARK_MTORC1_SIGNALING	200	4	0.00431	0.0239	9	4.81E-08	2.40E-06	NA	NA	NA	NA	NA	NA	3	0.00759	0.0237	NA	NA	NA	3	0.00567	0.0258
HALLMARK_KRAS_SIGNALING_UP	200	NA	NA	NA	4	0.00587	0.0183	8	3.31E-08	1.65E-06	NA	NA	NA	NA	NA	NA	6	2.70E-05	0.000112	NA	NA	NA
HALLMARK_P53_PATHWAY	200	NA	NA	NA	5	0.00078	0.00392	3	0.0128	0.0427	9	5.10E-08	1.28E-06	3	0.00759	0.0237	9	7.72E-09	5.51E-08	NA	NA	NA
HALLMARK_INTERFERON_ALPHA_RESPONSE	200	NA	NA	NA	NA	NA	NA	NA	NA	NA	4	0.00043	0.00305	6	4.47E-08	1.12E-06	5	9.71E-06	4.86E-05	NA	NA	NA
HALLMARK_INTERFERON_GAMMA_RESPONSE	200	NA	NA	NA	NA	NA	NA	4	0.00145	0.0145	7	8.57E-06	0.000143	7	1.62E-07	2.70E-06	11	1.81E-11	2.26E-10	5	3.14E-05	0.000393
HALLMARK_TNFA_SIGNALING_VIA_NFKB	200	NA	NA	NA	NA	NA	NA	4	0.00145	0.0145	14	2.10E-14	1.05E-12	8	7.13E-09	3.57E-07	32	4.47E-47	2.24E-45	NA	NA	NA
HALLMARK_INFLAMMATORY_RESPONSE	200	6	5.34E-05	0.00089	5	0.00078	0.00392	3	0.0128	0.0427	4	0.00601	0.0177	3	0.00759	0.0237	13	2.82E-14	7.06E-13	4	0.00048	0.00339
GO_SECRETION	1618	28	3.77E-13	2.85E-09	24	3.28E-09	2.26E-06	21	2.49E-10	1.89E-06	NA	NA	NA	NA	NA	NA	NA	NA	NA	NA	NA	NA
GO_EXOCYTOSIS	914	21	2.60E-12	6.56E-09	21	1.47E-11	1.24E-08	11	1.37E-05	0.00266	NA	NA	NA	NA	NA	NA	NA	NA	NA	11	6.97E-07	0.000132
GO_MYELOID_LEUKOCYTE_ACTIVATION	662	15	5.20E-09	7.88E-06	23	2.48E-16	1.88E-12	10	4.90E-06	0.00137	NA	NA	NA	NA	NA	NA	NA	NA	NA	NA	NA	NA
GO_ORGANIC_ACID_METABOLIC_PROCESS	1183	NA	NA	NA	26	1.14E-13	2.82E-10	NA	NA	NA	NA	NA	NA	NA	NA	NA	NA	NA	NA	NA	NA	NA
GO_ORGANIC_ACID_BIOSYNTHETIC_PROCESS	368	NA	NA	NA	14	7.24E-11	5.48E-08	NA	NA	NA	NA	NA	NA	NA	NA	NA	NA	NA	NA	NA	NA	NA
GO_RIBOSOME_BIOGENESIS	310	NA	NA	NA	NA	NA	NA	NA	NA	NA	14	8.20E-12	1.77E-09	NA	NA	NA	NA	NA	NA	NA	NA	NA
GO_CYTOPLASMIC_TRANSLATION	103	NA	NA	NA	NA	NA	NA	NA	NA	NA	11	1.32E-13	3.22E-11	NA	NA	NA	NA	NA	NA	NA	NA	NA
GO_PROTEIN_TARGETING_TO_MEMBRANE	207	NA	NA	NA	NA	NA	NA	NA	NA	NA	24	2.59E-29	2.45E-26	NA	NA	NA	NA	NA	NA	NA	NA	NA
GO_APOPTOTIC_PROCESS	1994	NA	NA	NA	21	8.66E-06	0.00179	NA	NA	NA	31	4.02E-12	9.22E-10	16	1.74E-06	0.000286	28	2.29E-12	1.02E-09	17	6.54E-08	1.90E-05
GO_RESPONSE_TO_BIOTIC_STIMULUS	1615	NA	NA	NA	NA	NA	NA	20	1.60E-09	6.05E-06	NA	NA	NA	23	5.39E-14	4.08E-10	24	3.54E-11	7.25E-09	17	2.97E-09	1.41E-06
GO_RESPONSE_TO_TYPE_I_INTERFERON	99	NA	NA	NA	NA	NA	NA	NA	NA	NA	NA	NA	NA	7	1.24E-09	1.57E-06	NA	NA	NA	NA	NA	NA
GO_RESPONSE_TO_OXYGEN_CONTAINING_COMPOUN	1714	NA	NA	NA	NA	NA	NA	NA	NA	NA	NA	NA	NA	15	1.33E-06	0.000241	32	1.41E-17	5.36E-14	NA	NA	NA
GO_REGULATION_OF_IMMUNE_SYSTEM_PROCESS	1637	NA	NA	NA	21	3.92E-07	0.000165	15	9.15E-06	0.00213	27	3.28E-11	6.38E-09	NA	NA	NA	28	1.93E-14	3.65E-11	19	5.52E-11	4.64E-08
GO_CYTOKINE_MEDIATED_SIGNALING_PATHWAY	807	NA	NA	NA	NA	NA	NA	11	4.26E-06	0.00124	NA	NA	NA	13	7.42E-09	6.25E-06	20	2.19E-13	2.08E-10	NA	NA	NA

K: number of genes in each gene set; k: number of genes in overlap. Results with FDR q-value < 0.05 are only shown.

Supplementary Table 2. List of antibodies and dyes used in histological staining

Primary antibodies				
	Supplier	Conjugated	Cat. #	Incubation
CD11b/c	Abcam	N/A	ab 1211	10ug/ml, 1h, RT
CD45	Biolegend	Pacific Blue	202226	2 in 100, 1h, RT
CD45	Biolegend	FITC	202205	4 in 100, 1h, RT
CD68	BMA Biomedicals	N/A	T-3003	0.5 in 100, 1h RT
Secondary antibodies				
	supplier	Conjugated	Cat. #	
Histofine® Simple StainTM MAX PO (M)	NICHIREI BIOSCIENCE	Peroxidase	424132	1h, RT
Fluorescein/Oregon Green Polyclonal antibody	Thermo fisher	Alexa Fluor 488	A-11096	0.5 in 100, 1h RT
FITC-conjugated				
Dye				
	Supplier		Cat. #	
Simple stain DAB solution	NICHIREI BIOSCIENCE		415172	5-10min, RT

Supplementary Table 3. List of antibodies and dyes used in flowcytometry analysis

A. List of antibodies used for staining ASCs

Antibody	Conjugated	Supplier	Clone	Dilution	Laser	Filter	Cat. #
Negative marker							
CD14	FITC	BD	M5E2	1in 5	Blue(488nm)	530/30	555397
CD34	PE	Santa Cruz	ICO115	1 in 5	Blue(488nm)	575/25	sc-7324
CD45	APC-Fire	Biolegend	HI30	1 in 20	Red(633nm)	780/60	304062
HLA-DR	APC	Biolegend	L243	1 in 20	Red(633nm)	660/20	307610
CD19	FITC	Biolegend	4G7	1 in 20	Blue(488nm)	530/30	392508
Positive marker							
CD44	PE	Biolegend	IM7	1 in 80	Blue(488nm)	575/25	103007
CD73	FITC	Biolegend	AD2	1 in 20	Blue(488nm)	530/30	344016
CD90	FITC	Biolegend	5.00E+10	1 in 20	Blue(488nm)	530/30	328108
CD105	PE	BD	266	1 in 20	Blue(488nm)	575/25	560839

B. Panel for myeloid cells and B cell analysis

Antibody	Conjugated	Supplier	Clone	Dilution	Laser	Filter	Cat. #
Biotin	PE-dazzle streptavidin	Biolegend		1 in 200	Blue(488nm)	610/20	405248
CD45RA	APC-Cy7	BD	OX-33	1 in 50	Red(633nm)	780/60	561624
CD161	PE	Biolegend	3.2.3.	1 in 50	Blue(488nm)	575/25	205604
CD3	Biotin	Thermo fisher	G4.18	1 in 50			13-0030-82
CD32	N/A	BD	D34-485	1 in 50			550271
CD43	PE-Cy7	Biolegend	W3/13	1 in 50	Blue(488nm)	780/60	202816
CD45	Pacific Blue	Biolegend	OX-1	1 in 50	Violet(405nm)	450/50	202226
HIS48	FITC	Thermo fisher	HIS48	1 in 50	Blue(488nm)	530/30	11-0570-82
Live-dead	Zombie Aqua	Biolegend		1 in 100	Violet(405nm)	525/50	423102

C. Panel for T cell analysis

Antibody	Conjugated	Supplier	Clone	Dilution	Laser	Filter	Cat. #
CD25	PE	Biolegend	OX-39	1 in 100	Blue(488nm)	575/25	12-0390-82
CD3	BV605	BD	1F4	1 in 50	Violet(405nm)	610/20	563949
CD32	N/A	BD	D34-485	1 in 50			550271
CD4	FITC	Biolegend	OX-35	1 in 100	Blue(488nm)	530/30	203305
CD45	Pacific Blue	Biolegend	OX-1	1 in 50	Violet(405nm)	450/50	202226
CD8a	PerCP	Biolegend	OX8	1 in 25	Blue(488nm)	660/20	201712
Foxp3	APC	Biolegend	FJK16-s	1 in 50	Red(633nm)	660/20	17-5773-

							82
Live-dead	Zombie Aqua	Biolegend		1 in 100	Violet(405nm)	525/60	423102

D. Panel for Ki 67 analysis in Tregs

Antibody	Conjugated	Supplier	Clone	Dilution	Laser	Filter	Cat. #
CD25	PE	Biolegend	OX-39	1 in 100	Blue(488nm)	575/25	12-0390-82
CD3	BV605	BD	1F4	1 in 50	Violet(405nm)	610/20	563949
CD32	N/A	BD	D34-485	1 in 50			550271
CD4	PE-Cy7	Biolegend	W3/25	1 in100	Blue(488nm)	780/60	201516
CD45	Pacific Blue	Biolegend	OX-1	1 in 50	Violet(405nm)	450/50	202226
CD8a	PerCP	Biolegend	OX8	1 in 25	Blue(488nm)	660/20	201712
Foxp3	APC	Biolegend	FJK16-s	1 in 50	Red(633nm)	660/20	17-5773-82
Ki67	FITC	Thermo fisher	SoLA15	1 in 100	Blue(488nm)	530/30	2040334
Live-dead	Zombie Aqua	Biolegend		1 in 100	Violet(405nm)	525/60	423102

E. Panel for CD45+ CD11b/c+ DiD+ cells subset sorting from spleen

Antibody	Conjugated	Supplier	Clone	Dilution	Laser	Filter	Cat. #
CD11b/c	FITC	Biolegend	OX-42	1 in 200	Blue(488nm)	530/30	201805
CD32	N/A	BD	D34-485	1 in 50			550271
CD45	Pacific Blue	Biolegend	OX-1	1 in 50	Violet(405nm)	450/50	202226

F. Panel for M1 and M2 macrophage subsets sorting

Antibody	Conjugated	Supplier	Clone	Dilution	Laser	Filter	Cat. #
Biotin	CD11b/c	Biolegend	OX-12	1 in 100			201803
CD3	BV605	BD	1F4	1 in 50	Violet(405nm)	610/20	563949
CD32	N/A	BD	D34-485	1 in 50			550271
CD45	Pacific Blue	Biolegend	OX-1	1 in 50	Violet(405nm)	450/50	202226
HIS48	FITC	Thermo fisher	HIS48	1 in 50	Blue(488nm)	530/30	11-0570-82
Live-dead	Zombie Aqua	Biolegend		1 in 100	Violet(405nm)	525/50	423102

G. Panel for CD4+ T cell sorting from tibia

Antibody	Conjugated	Supplier	Clone	Dilution	Laser	Filter	Cat. #
CD3	BV605	BD	1F4	1 in 50	Violet(405nm)	610/20	563949
CD32	N/A	BD	D34-485	1 in 50			550271
CD45	Pacific Blue	Biolegend	OX-1	1 in 50	Violet(405nm)	450/50	202226
Live-dead	Zombie Aqua	Biolegend		1 in 100	Violet(405nm)	525/50	423102

Supplementary Table 4. List of primers used to detect mRNAs

Rps18	Fw	AAGTTTCAGCACATCCTGCGAGTA
	Rv	TTGGTGAGGTCAATGTCTGCTTTC
IL-10	Fw	CAGACCCACATGCTCCGAGA
	Rv	CAAGGCTTGGCAACCCAAGTA
TGFβ1	Fw	CATTGCTGTCCCGTGCAGA
	Rv	AGGTAACGCCAGGAATTGTTGCTA
IL-6	Fw	ATTGTATGAACAGCGATGATGCAC
	Rv	CCAGGTAGAAACGGAACTCCAGA
TNFα	Fw	TTCCAATGGGCTTTCGGAAC
	Rv	AGACATCTTCAGCAGCCTTGTGAG
Ccr2	Fw	TGTGAGGCTCATCTTTGCCATC
	Rv	CACCTGCATGGCCTGGTCTA

Fw, forward ; Rv, reverse
