# Supplementary material for: Mesenchymal stem cells exert renoprotection via extracellular vesicle-mediated modulation of M2 macrophages and spleen-kidney network
Source: Commun Biol. 2022 Jul 28;5:753. doi: 10.1038/s42003-022-03712-2 (PMC9334610; doi:10.1038/s42003-022-03712-2)
Supplement: Supplementary file 3 — Description of Additional Supplementary Files [file 42003_2022_3712_MOESM3_ESM.pdf]

Description of Additional Supplementary Files

File name: Supplementary Data
Description: The source data underlying most graphs and charts used in this manuscript.

File name: Supplementary Movie 1
Description: The coculture of DiD labeled ASC and macrophages.

File name: Supplementary Movie 2
Description: The behavior of EV+ leukocytes in spleen.
