# Supplementary material for: Mesenchymal stem cells exert renoprotection via extracellular vesicle-mediated modulation of M2 macrophages and spleen-kidney network
Source: Commun Biol. 2022 Jul 28;5:753. doi: 10.1038/s42003-022-03712-2 (PMC9334610; doi:10.1038/s42003-022-03712-2)
Supplement: Supplementary file 7 — Reporting Summary [file 42003_2022_3712_MOESM7_ESM.pdf]

nature portfolio

Corresponding author(s): Kazuhiro Furuhashi

Last updated by author(s): June 16, 2022

Reporting Summary

Nature Portfolio wishes to improve the reproducibility of the work that we publish. This form provides structure for consistency and transparency in reporting. For further information on Nature Portfolio policies, see our Editorial Policies and the Editorial Policy Checklist.

Statistics

For all statistical analyses, confirm that the following items are present in the figure legend, table legend, main text, or Methods section.

n/a	Confirmed	
☐	☒	The exact sample size (n) for each experimental group/condition, given as a discrete number and unit of measurement
☐	☒	A statement on whether measurements were taken from distinct samples or whether the same sample was measured repeatedly
☐	☒	The statistical test(s) used AND whether they are one- or two-sided<br>*Only common tests should be described solely by name; describe more complex techniques in the Methods section.*
☒	☐	A description of all covariates tested
☒	☐	A description of any assumptions or corrections, such as tests of normality and adjustment for multiple comparisons
☐	☒	A full description of the statistical parameters including central tendency (e.g. means) or other basic estimates (e.g. regression coefficient) AND variation (e.g. standard deviation) or associated estimates of uncertainty (e.g. confidence intervals)
☒	☐	For null hypothesis testing, the test statistic (e.g. F, t, r) with confidence intervals, effect sizes, degrees of freedom and P value noted<br>*Give P values as exact values whenever suitable.*
☒	☐	For Bayesian analysis, information on the choice of priors and Markov chain Monte Carlo settings
☒	☐	For hierarchical and complex designs, identification of the appropriate level for tests and full reporting of outcomes
☒	☐	Estimates of effect sizes (e.g. Cohen's d, Pearson's r), indicating how they were calculated

Our web collection on statistics for biologists contains articles on many of the points above.

Software and code

Policy information about availability of computer code

Data collection	Static fluorescence micrographs were acquired with a Nikon TiE-A1R laser scanning confocal microscope, dynamic in vivo images were acquired with a Nikon A1RMP microscope, and dynamic observations in culture exposures were acquired with a Keyence BZ-X800 analyzer. For qPCR, RNA from sorted cells was extracted using RNeasy Plus Micro Kits. Libraries for RNA-seq were prepared using SMART-Seq v4 Ultra Low Input RNA Kits for Sequencing. For flow cytometry, a BD FACS SORP Aria II was used. EVs were obtained by L-80XP ultracentrifuged using a W32Ti rotor.
Data analysis	Statistical analysis was performed using GraphPad Prism version9.0.The flowcytometry data was analyzed with FlowJo v10.The sequencing reads were aligned to the rn6 reference genome using STAR (v2.5.3)32. Reads in each refSeq gene were counted with HTSeq (v0.6.0) using the intersection-strict model33. The edgeR package in R was used to identify the differentially expressed genes.The acquired fluorescence micrographs were analyzed by Imaris ver7.6.

For manuscripts utilizing custom algorithms or software that are central to the research but not yet described in published literature, software must be made available to editors and reviewers. We strongly encourage code deposition in a community repository (e.g. GitHub). See the Nature Portfolio guidelines for submitting code & software for further information.

Data

Policy information about availability of data

All manuscripts must include a data availability statement. This statement should provide the following information, where applicable:

- Accession codes, unique identifiers, or web links for publicly available datasets
- A description of any restrictions on data availability
- For clinical datasets or third party data, please ensure that the statement adheres to our policy

RNA sequencing data are available in the Gene Expression Omnibus under accession number GSE179301. RNAseq data has not been deposited to any online databases, but can be made available upon request.

Human research participants

Policy information about studies involving human research participants and Sex and Gender in Research.

Reporting on sex and gender	*Use the terms sex (biological attribute) and gender (shaped by social and cultural circumstances) carefully in order to avoid confusing both terms. Indicate if findings apply to only one sex or gender; describe whether sex and gender were considered in study design whether sex and/or gender was determined based on self-reporting or assigned and methods used. Provide in the source data disaggregated sex and gender data where this information has been collected, and consent has been obtained for sharing of individual-level data; provide overall numbers in this Reporting Summary. Please state if this information has not been collected. Report sex- and gender-based analyses where performed, justify reasons for lack of sex- and gender-based analysis.*
Population characteristics	Human perirenal adipose tissue was obtained from living donors undergoing kidney transplantation after obtaining their informed consent.
Recruitment	After giving their agreement, the donors provided fat to be discarded during the transplant procedure.
Ethics oversight	All experiments using human tissue samples were approved by the ethical committee at the Nagoya University Medical School (approval number 2005-0347-5) and the guidelines of the Declaration of Helsinki.

Note that full information on the approval of the study protocol must also be provided in the manuscript.

Field-specific reporting

Please select the one below that is the best fit for your research. If you are not sure, read the appropriate sections before making your selection.

☒ Life sciences ☐ Behavioural & social sciences ☐ Ecological, evolutionary & environmental sciences

For a reference copy of the document with all sections, see nature.com/documents/nr-reporting-summary-flat.pdf

Life sciences study design

All studies must disclose on these points even when the disclosure is negative.

Sample size	Sample sizes were chosen empirically based on our preliminary experiments, using this animal model, to assure adequate statistical power. All experiments were repeated independently,<br>Determination of treatment response: n=7-8 per group. Cell tracking: n=3 for imaging, n=7 for flowcytometory. In vitro asssay: n=3-5.
Data exclusions	No data was excluded.
Replication	Allexperiments in this study was independently replicated, with a minimum of two biological and technical replicates as indicated in the text or corresponding figure legends.
Randomization	All animals were randomly allocated into each experimental groups for all animal study.
Blinding	Investigators were not blinded to allocation during experiments and outcomes assessment. However, histological score was performed from one blinded score. Blood biochemistry analysis was performed by a blinded third-party.

Reporting for specific materials, systems and methods

We require information from authors about some types of materials, experimental systems and methods used in many studies. Here, indicate whether each material, system or method listed is relevant to your study. If you are not sure if a list item applies to your research, read the appropriate section before selecting a response.

Materials & experimental systems

n/a	Involved in the study	
☐	☒	Antibodies
☒	☐	Eukaryotic cell lines
☒	☐	Palaeontology and archaeology
☐	☒	Animals and other organisms
☒	☐	Clinical data
☒	☐	Dual use research of concern

Methods

n/a	Involved in the study	
☒	☐	ChIP-seq
☐	☒	Flow cytometry
☒	☐	MRI-based neuroimaging

Antibodies

Antibodies used	List of antibodies and dyes used in histological staining is in Table S1.<br>List of antibodies and dyes used in flow cytometric analysis is in Table S2.
Validation	All antibodies used are commercially available and have been validated tor the application used by the manufacturer. Specific references for each antibody can be found on the manufactures website. Appropriate controls were use d tor all immunostaining.

Animals and other research organisms

Policy information about studies involving animals; ARRIVE guidelines recommended for reporting animal research, and Sex and Gender in Research

Laboratory animals	WKY/NCrj rats were purchased from Charles River, Inc. (Yokohama, Japan). Female 7-12-week-old rats were used for the nephritis model.
Wild animals	This study did not involve any wild animals.
Reporting on sex	Female rats were used in the evaluation of proteinuria in urine storage because of the possibility of sperm contamination in male rats.
Field-collected samples	All rats were maintained on a 12-hour light/dark cycle with free access to standard diet and water, in accordance with the National Institutes of Health Guide for the Care and Use of Laboratory Animals.
Ethics oversight	All animal experimental protocols were conducted in accordance with the guidelines of the Institutional Animal Ethics Committee of Nagoya University Graduate School of Medicine (Approval number 20377).

Note that full information on the approval of the study protocol must also be provided in the manuscript.

Flow Cytometry

Plots

Confirm that:

☒ The axis labels state the marker and fluorochrome used (e.g. CD4-FITC).

☒ The axis scales are clearly visible. Include numbers along axes only for bottom left plot of group (a 'group' is an analysis of identical markers).

☒ All plots are contour plots with outliers or pseudocolor plots.

☒ A numerical value for number of cells or percentage (with statistics) is provided.

Methodology

Sample preparation	Tissue samples were cut into small fragments using a razor and suspended in media containing 1 mg/mL collagenase type I (Worthington Biochemical Corporation, Lakewood, NJ, USA) and 1 mg/ml DNase I (Merck, Darmstadt, Germany). Samples in the dissociation solution were incubated at 37 °C and gently agitated for 30 min. The tissue fragments were then passed several times through an 18-gauge needle using 1 ml syringes and filtered through a 100 µm strainer. The collagenase reaction was stopped by adding PBS with 5 mM EDTA (Thermo Fisher Scientific, MA, USA). The isolated cells were pelleted at 400 g at 4 °C. The red blood cells in the samples were depleted with BD Pharm Lyse (BD, San Jose, CA, USA). The cells were blocked with anti-CD32 antibody (1 in 50 BD, 550271) and stained with the labeled monoclonal antibodies listed in Table S2.
Instrument	Cells were sorted and analyzed using a FACS SORP Aria II (BD Bioscience, Tokyo, Japan).
Software	FlowJo software (v10) (BD Bioscience, Tokyo, Japan) was used.

Cell population abundance	The purity of the sorted cells was determined for each group of sorted cells using the same sorted instrument, and the purity was over 95%.
Gating strategy	Gating strategy was shown in supplementary Figure 2.

☒ Tick this box to confirm that a figure exemplifying the gating strategy is provided in the Supplementary Information.
